# Measuring Perseverance and Passion in Distance Education Students: Psychometric Properties of the Grit Questionnaire and Associations With Academic Performance

**DOI:** 10.3389/fpsyg.2020.563585

**Published:** 2020-12-14

**Authors:** Kate M. Xu, Celeste Meijs, Hieronymus J. M. Gijselaers, Joyce Neroni, Renate H. M. de Groot

**Affiliations:** Faculty of Educational Sciences, Open University of the Netherlands, Heerlen, Netherlands

**Keywords:** distance education, grit, adult students, psychometric validation, academic performance

## Abstract

With modern technological advances, distance education has become an increasingly important education delivery medium for, for example, the higher education provided by open universities. Among predictive factors of successful learning in distance education, the effects of non-cognitive skills are less explored. Grit, the dispositional tendency to sustain trait-level passion and long-term goals, has raised much research interest and gained importance for predicting academic achievement. The Grit Questionnaire, measuring Perseverance of Effort and Consistency of Interests, has been shown to be a reliable instrument in traditional university student populations. However, the measurement and predictive validity of this questionnaire is still unknown for adult distance education university students who differ from traditional students in various ways (e.g., having a wider range of student ages). Based on a sample of 2,027 students from a distance education university, this study assessed the psychometric properties of the two-factor structure grit measured by the Grit Questionnaire. The findings suggest that the short form of the Grit Questionnaire is a potentially useful assessment tool for measuring the grit construct for distance learning higher education and that the Consistency of Interests factor is especially relevant to consider the improvement of learning performance for distance education in terms of courses credit and exam attempts. The measurement precision of the Perseverance of Effort factor, however, should be improved in future research to provide higher measurement accuracy and broader item coverage.

## Introduction

With modern technological advances, distance education has become an increasingly important education delivery medium as a way of lifelong and continuous learning (Eurydice, 2011). Among predictive factors of learning in distance education, the effects of non-cognitive skills are less explored (Aparicio et al., 2017). The role of grit—perseverance and passion for long-term goal pursuit—might especially be important for adult distance education students, because these students are not only older than typical university students but also are more likely to have a full-time or part-time job and/or family responsibilities. Given the multitude of personal and societal responsibilities, possessing grit, being committed to both effort and interest toward their study might be especially critical for their academic performance. To study the relationship between grit and relevant outcomes in adult distance education, it is important that the instrument measuring grit is reliable and valid. To our knowledge, no previous studies have examined the psychometric properties of a questionnaire measuring grit for adult distance education students.

Recently, the power of non-cognitive character strengths, in particular grit has been the subject of widespread research interest (Schmitt, 2012; West et al., 2016). This line of research has led to government interests to include perseverance as one of the non-cognitive factors critical for study success (Shechtman et al., 2013). Schools are also starting to incorporate assessment of such character traits as part of the curriculum (Sparks, 2015; Zernike, 2016). Duckworth et al. (2007) defined grit as a trait-level passion, Perseverance of Effort, and Consistency of Interests needed to attain long-term goals. The Grit Questionnaire has been used to predict not only achievement in the academic domain (Duckworth et al., 2007; Eskreis-Winkler et al., 2014) but also other important outcomes such as cognitive functions (Abuhassàn and Bates, 2015; Li et al., 2018b), self-efficacy (Wolters and Hussain, 2015), academic engagement (Datu et al., 2016), and subjective well-being (Datu et al., 2016; Li et al., 2018a; Disabato et al., 2019), which also play important roles in academic performance (Howell, 2009). Recent research findings in neuroscience have also advanced the understanding of the grit measurement by demonstrating associations between grit and relevant neuroanatomical correlates (Wang et al., 2017, 2018).

Despite the rapidly increasing number of studies that are being conducted on grit and its correlates, important gaps remain regarding the generalizability of the measurement and the predictive validity of the Grit Questionnaire in other student populations. In particular, much of the previous literature has focused on full-time students, in particular those in secondary and higher education. Little is known in adult distance education regarding whether grit can be reliably measured across student demographic background such as prior level of education, age, and gender groups and if its relationship with achievement holds true for learners of alternative educational settings such as those (Hwang et al., 2017, see also Aparicio et al., 2017).

The current study aims to psychometrically evaluate the Grit Questionnaire in a sample of adult distance education students attending a large distance education University, as well as to examine its association with academic performance. In the following, we review previous psychometric studies in students of higher education and general adult population that examined the factor structure of the Grit scale and the association between grit and academic performance (Arslan et al., 2013; Datu et al., 2016).

### Psychometric Studies of Grit Questionnaire

#### The Factor Structure of Grit

**FIGURE 1:**
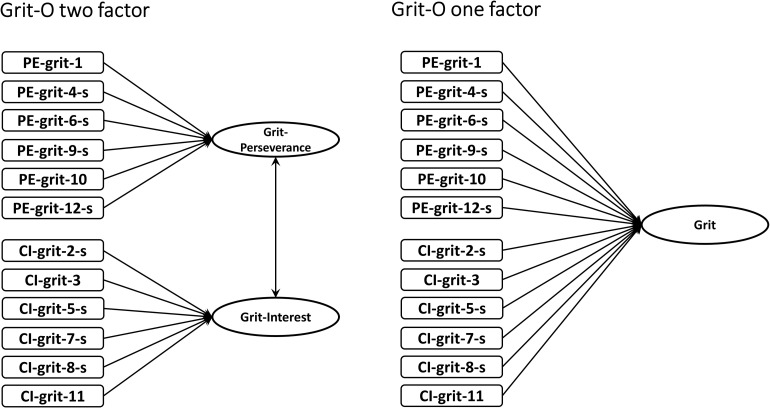
Psychological construct of the 12-item original Duckworth Grit Questionnaire (one-factor and two-factor). The letter “s” denotes the short-version of the Grit Questionnaire items. See Table 1 for item wordings.

The original grit scale (Grit-O) consisted of 12 items measuring two dimensions: six measuring the Perseverance of Effort dimension and six measuring the Consistency of Interests dimension (see latent variable representation in Figure 1). The Perseverance of Effort dimension describes the extent to which an individual sustains continued effort in the face of challenges, whereas the Consistency of Interests dimension focuses on the sustainability of passion—the interest the person maintains over time. The Grit Questionnaire was initially developed and validated in a sample of 1,545 adults (age > 25, Study 1; Duckworth et al., 2007). Although factor analysis of an initial pool of items in Study 1 suggested two dimensions (i.e., Perseverance of Effort and Consistency of Interests), the authors combined the items from the two scales into one single total score in subsequent analyses. This has raised much debate in terms of the structure of the grit construct (Credé et al., 2017; Credé, 2018). Since the two Grit dimensions were shown as separate factors, combining the two into a single factor would imply a unidimensional construct misrepresenting the underlying structure. Credé et al. (2017) showed in a meta-analysis that the two factors were moderately correlated (*r* = 0.44), and this was confirmed by a more recent meta-analysis by Guo et al. (2019) (*r* = 0.43). If the one-factor structure holds true, the two factors would have a correlation approaching 1, which is not the case.

#### The Short Version of the Grit Questionnaire

Following the development of the Grit-O, the authors developed a shortened version based on eight items from the full version (Grit-S; Duckworth and Quinn, 2009). The Grit-S has been shown to possess better psychometric properties than the Grit-O and hence, most empirical studies on grit have utilized the Grit-S questionnaire (e.g., Datu et al., 2016; Hwang et al., 2017; Wyszyńska et al., 2017). Wyszyńska et al. (2017) validated the Polish version of the Grit-S scale in 270 adults aged 18–34 in the general population. The two-factor model was found to fit the data better than the one-factor unidimensional model. The two-factor Grit structure was also supported in Philippine (*n* = 220; Datu et al., 2016) and US undergraduate students (*n* = 336; Muenks et al., 2017).

In a sample of female adult distance education students in Korea (Hwang et al., 2017), based on the Grit-O scale, the authors studied the relationship between grit, maladjustment (e.g., difficulties in finishing class assignments), and achievement. However, the authors did not perform a psychometric evaluation of the Grit items, thus it is still unknown which factor structure holds for adult distance education students. Therefore, in the present study, the factor structures of both the Grit-O and Grit-S scales were investigated in a sample of distance education in both male and female students, participating in adult online distance education.

#### Measurement Invariance of Grit

Since grit is considered a dispositional trait, the psychometric properties of the grit construct may be subject to gender and age differences as shown in personality research (Soto et al., 2011; see also Xu et al., 2017). Adult distance education student population has a more diverse range of age; it is thus important to assess whether measurement of the Grit Questionnaire holds invariant across age. Previous research examining psychometric properties of the Grit Questionnaires in adult populations has supported measurement invariance in relation to gender (e.g., Duckworth and Quinn, 2009; Wyszyńska et al., 2017). However, there is still limited research examining whether the Grit Questionnaire is measurement invariant across ages in adult or adult distance education populations.

Many studies have found a positive association between grit and level of education obtained (Duckworth et al., 2007; Palczyńska and Świst, 2018). Since distance education students often have diverse educational backgrounds, it is important to examine whether measurement properties of the Grit Questionnaire holds equal across prior educational levels. However, most of the previous research focused on and confirmed measurement invariance of level of education by comparing samples currently receiving secondary (e.g., Areepattamannil and Khine, 2017) and/or university education (e.g., Datu et al., 2016). The extent to which previous educational levels affect measurement properties of the Grit Questionnaire in distance education student is still unknown.

### The Association Between Grit and Academic Performance

Of the two factors measured in the Grit Questionnaire, the Perseverance of Effort factor has particularly shown to be predictive of achievement-related outcomes. In the meta-analysis of Credé et al. (2017), this factor was higher correlated with academic performance measured by grade point average (*r* = 0.20) than the correlation for the Consistency of Interests factor (*r* = 0.08). In more recent studies, Palczyńska and Świst (2018) reported a positive association (*b* = 0.07) between Consistency of Interests and educational attainment (highest level of education obtained) measured in a sample of adults (aged 18–69, *N* = 4,355) from Poland. In a study based on traditional higher education students by Muenks et al. (2017), Perseverance of Effort predicted grade point average (*b* = 0.17). Perseverance of Effort was also found to positively predict grade-point average in another study based on US undergraduate students (*n* = 209, *b* = 0.22; Akos and Kretchmar, 2017); however, neither factors of Grit predicted course credit. Few studies investigated the predictive effect of grit on academic performance in adult distance education students, except for one study based on a sample of adult distance education students in Korea (Hwang et al., 2017). The authors found that neither Consistency of Interests nor Perseverance of Effort directly predicted GPA; however, Perseverance of Effort predicted academic maladjustment (e.g., difficulties in finishing class assignments) which in turn predicted grade-point average. The current study further aimed to investigate the predictive effects of grit factors on academic performance in a sample of adult distance education students.

The present study has primarily focused on the literature of the Grit Questionnaire developed by Duckworth et al. (2007). However, it is important to acknowledge that the Duckworth Grit Questionnaire is not the only theoretical framework of the grit construct. In particular, a triarchic model of grit has recently been developed and empirically supported to allow more culturally diverse adaptation of grit (Datu et al., 2017, 2018, 2020). Passion has also been suggested as an informative aspect of grit (Jachimowicz et al., 2018; see also Guo et al., 2019). Furthermore, the validity of the Grit Questionnaire has been under much debate, in particular regarding its uniqueness in relation to other relevant personality trait such as conscientiousness and self-control (Credé, 2018, 2019; also see Guo et al., 2019). The present investigation primary attempts to examine not all but several aspects of the psychometric properties of the Duckworth Grit Questionnaire.

Based on a large sample of adult distance education university students, the present investigation addresses the gaps in the literature of the Duckworth Grit Questionnaire in terms of its factor structure (one factor vs. two factors), item characteristics, and measurement invariance related to age, gender, and educational level, as well as its predictive validity for academic achievement through the following three research aims, using sophisticated analytical approach based on item response theory (IRT).

## Research Questions

1. Evaluating the psychometric properties of the original and short versions of the Grit Questionnaire in a distance education setting. It is expected that the two-factor structure model fits the data better in comparison with the one-factor structure model, and that the factor structure based on Grit-S fits the data better than on Grit-O. However, in terms of item-level properties, limited previous research has examined aspects such as item discrimination indices and threshold levels (difficulty).
2. Assessing the measurement invariance of grit construct across gender, age, and educational levels. Based on previous research, psychometric properties of items measuring grit will be invariance in terms of gender; however, no specific hypothesis is made regarding age and prior educational levels due to lack of previous empirical research.
3. Investigating the predictive validity of the Grit Questionnaire with regard to academic performance (course grades, course credits, and exam attempts in a distance education setting). Although Perseverance of Effort has been found to be a more consistent predictor of achievement (Credé et al., 2017) in traditional higher education, in adult samples, Consistency of Interests rather than Perseverance of Effort was associated with educational attainment (Palczyńska and Świst, 2018). It is possible that for distance education students, also Consistency of Interests rather than Perseverance of Effort is more predictive of achievement.

## Materials and Methods

### Design, Participants, and Procedure

The sample for the present study was drawn from a larger study—the Adult Learning Open University Determinants (ALOUD) study. The ALOUD study is an investigation of biological and psychological determinants of study success possibly affecting academic performance in adult students participating in adult distance education, the Open University of the Netherlands (OUNL; Neroni et al., 2015). Previous publications on the ALOUD study can be found here (Gijselaers et al., 2015, 2016a,b,c, 2017; Meijs et al., 2019). All new students attending the OUNL registered in the period of August 2012 to August 2013 (*N* = 4,945) were approached to participate in ALOUD. Since OUNL is the only distance education university in the Netherlands, participants included in our study represent well the population under investigation. At baseline, an online survey lasting 45–60 min on average was administered regarding psychological, biological, and background variables using LimeSurvey^®^, version 1.92+ (LimeSurvey Project Team/Carsten Schmitz, 2012). During a 14-month follow-up, data regarding academic performance based on objective measures were also collected through the examination registration office. The 14-month period is in line with the subscription duration for a course at this university. More information about this cohort can be found in the data description paper of the ALOUD study (Neroni et al., 2015).

The analysis sample for the present study was based on participants with available data in variables measuring Grit and academic performance. The Grit Questionnaire was measured at baseline as part of the online survey. Of those who responded (*n* = 2,842) at baseline, 2,027 participants provided complete response to the Grit Questionnaire.

In the current sample, the age of participants ranged from 18 to 80, with the largest part (60%) being between 26 and 47 years old. Most participants already had a higher vocational or university/postgraduate degree (71%). There were more female students (62%, *n* = 1,253) than male students (38%, *n* = 774). At the time of the data collection, 88.3% of the participants held Dutch nationality (*n* = 1,790). Information on marital status was not collected by this study but 28.4% participants reported living with a partner (*n* = 560) and a further 35.2% living with a partner and children (*n* = 695). To control for potential confounding effects due to diverse demographic backgrounds, age, gender, and level of previous education were included as covariates in the predictive analysis of the current study.

### Measures

#### Grit

All 12 items of the Grit-O questionnaire (Table 1; translated and back translated by native speakers of both the native language in the student population as well as the English language) were assessed in the form of a 5-point Likert scale, ranging from “completely disagree” to “completely agree.” The internal consistency statistics measured by Cronbach’s Alpha were good for the Grit global scale (Grit-O *α* = 0.79; Grit-S *α* = 0.76), Perseverance of Effort dimension (Grit-O *α* = 0.67; Grit-S *α* = 0.62) and Consistency of Interests dimension (Grit-O *α* = 0.82; Grit-S *α* = 0.76).

**TABLE 1:** Descriptive statistics.

**Item**	**Dimension**	**Version**	**Mean**	**Skewness**	**Kurtosis**	**Item wording**
1	Perseverance	Original	3.91	–0.84	0.48	I have overcome setbacks to conquer an important challenge.
4	Perseverance	Original, short	3.49	–0.44	–0.43	Setbacks don’t discourage me.
6	Perseverance	Original, short	4.11	–0.69	0.44	I am a hard worker.
9	Perseverance	Original, short	3.84	–0.65	0.40	I finish whatever I begin^*a*^.
10	Perseverance	Original	3.69	–0.69	–0.31	I have achieved a goal that took years of work.
12	Perseverance	Original, short	3.91	–0.65	0.71	I am diligent.
2	Consistency	Original, short	2.69	0.37	–0.62	New ideas and projects sometimes distract me from previous ones.
3	Consistency	Original	3.34	–0.42	–0.51	My interests change from year to year.
5	Consistency	Original, short	3.52	–0.49	–0.34	I have been obsessed with a certain idea or project for a short time but later lost interest.
7	Consistency	Original, short	3.46	–0.45	–0.30	I often set a goal but later choose to pursue a different one.
8	Consistency	Original, short	3.55	–0.51	–0.45	I have difficulty maintaining my focus on projects that take more than a few months to complete.
11	Consistency	Original	3.26	–0.18	–0.74	I become interested in new pursuits every few months.

*^*a*^Item 9 was loaded on both dimensions in the revised Grit-S model.*

#### Academic Performance

Academic performance during 14 months after the study baseline were operationalized as total course credit, average course grade, and exam attempt.

##### Exam Attempt

This performance measure is based on whether a student attempted taking an exam. Within the 2,027 students who provided complete data on the Grit Questionnaire, 1,133 had made exam attempts, whereas the remaining 894 did not make an attempt during the 14-month follow-up. This is an important indicator of study progress because more than 50% of the responders in the investigated population did not complete any course after 14 months, and many of them reported not having started studying. This is normal at such universities (Gijselaers et al., 2017). Since a course grade can only be obtained after a successful exam attempt, it is highly relevant to investigate whether grit is related to exam attempt.

##### Course Credit

Course credit was the number of successfully completed study modules in 14 months (min = 1, max = 22; mean = 3.18, *SD* = 2.54). A course at this university consists of one or more modules. One module is equal to 4.3 European Credits in the European Credit Transfer System. The nominal study load for one module is approximately 120 study hours.

##### Course Grade

Average course grades were calculated across courses taken (min = 6, max = 10; mean = 7.16, *SD* = 0.90). A grade is a score between 1 and 10, with 10 being the best possible score.

Only the grades of passed exams were used to calculate the course grade, with six being the passing grade. From the 1,133 students who had made exam attempts, 952 were successful. The average course grade was available for those students. However, for the 181 students who failed the exam attempt, no information was available for their course grades nor course credits.

All learning performance measures were derived from objectively measured learning performance data provided by the exam registration office of this adult distance education university. The assessments of most courses measured in this study were timed computerized exams which students had to perform at one of the 21 study centers of this adult distance education University located throughout the country and part of a neighbor country (Belgium) having the same mother tongue.

## Analysis

### Model Specification

An IRT analytical approach was used for the present study, embedded in the framework of confirmatory factor analysis (CFA) and structural equation modeling (SEM) methods, in statistical software Mplus 7.11 (Muthén and Muthén, 2015), using weighted least squares estimator with theta parameterization (WLSMV; Muthén and Muthén, 2015). The IRT method affords appropriate treatment of the Likert scale format response data as categorical variables. Under Mplus MLSMV specification, data were modeled as ordered-categorical polytomous ratings via a probit regression link to the corresponding latent variables. These methods correspond to a graded response normal ogive IRT model with two parameters (Samejima, 1997). For each fitted model under whole sample or multiple group analysis specification, parameters associated with each included items were estimated in terms of error residuals, discrimination indices (factor loading), and four thresholds (difficulty) for each item corresponding to the five Likert scale categories. Factor variances and covariances were estimated for latent variables. Factor means were also estimated in the case of multiple group analysis models.

### Measurement Invariance

Multiple group analysis was used to examine the measurement invariance in relation to gender. Differential item functioning (DIF) analysis was used to examine whether there is measurement invariance related to age and prior education levels. For measurement invariance regarding gender, four multiple group models were estimated: configural invariance, weak invariance (discrimination indices/factor loadings), strong invariance (discrimination indices/factor loadings and thresholds), strict invariance (discrimination/factor loadings, thresholds, and uniqueness), and structural invariance (variance and covariance of latent constructs). The DIF refers to the presence of an association between the covariate and an item while controlling for the regression path from the covariate to the latent variable and the covariate. This indicates that the latent variable alone does not account for the relationship between the covariate and the item, thus a measurement bias is present. DIF is analogous to non-invariance of the item threshold and suggests response bias of the item (Kaplan, 2000). To assess DIF in relation to age and prior educational levels, we specified two models (Woods, 2009, also see Morin et al., 2013). In the DIF baseline model (saturated model), the path coefficients between the covariates and the latent variables were constrained to be zero, with the direct paths from the covariates to the items freely estimated. In the DIF comparison model (the more restrictive invariance model), the paths coefficients from the covariates to the items were fixed at zero, but the paths from the covariates to the latent factors are freely estimated. To evaluate the extent of measurement invariance based on multiple group and DIF analysis, goodness-of-fit indices are used to evaluate whether there is a decrease of model fit as the invariant constricts become more restrictive.

### Model Evaluation

Model goodness of fit was evaluated using a range of fit indices. Since the chi-square statistic is known to be highly sensitive to sample size (Marsh et al., 1988, 2005), a variety of sample-size-independent goodness-of-fit indices were also examined to assess the fit of the alternative models: the Root Mean Square Error of Approximation (RMSEA), the Tucker-Lewis Index (TLI), and the Comparative Fit Index (CFI; Fan et al., 1999; Hu and Bentler, 1999; Yu, 2002; Marsh et al., 2004). The TLI and CFI vary along a 0–1 continuum and values greater than 0.90 and 0.95 typically reflect an acceptable and excellent fit to the data. RMSEA values of less than 0.06 and 0.08 indicate a close fit and an acceptable fit to the data, respectively. In terms of model comparisons for multiple-group analyses, a restrictive model is preferred if the change in model fit indices is not significantly inferior to those of the less restrictive model. For RMSEA, the change should be less than 0.015 (Chen, 2007). For CFI and TLI, the change should be less than 0.01 (Cheung and Rensvold, 2001; Chen, 2007). The chi-square difference tests for model comparison were computed with the DIFFTEST function in Mplus with the WLSMV estimator (Muthén and Muthén, 2015).

## Results

### Factor Structure of Grit

Two separate IRT-CFA models were fitted for both the Grit-O and Grit-S scales. The first model was based on the structure of a single first-order factor, assuming unidimensionality of the Grit construct (Figure 1). In this model, all items were loaded on one factor (Model M1 for Grit-O, and Model M3 for Grit-S, Table 2). The second model tested the two-factor structure, with Perseverance of Effort and Consistency of Interests as separate dimensions (Figure 1; Model M2 for Grit-O, Model M4 for Grit-S, Table 2). As expected, the two-factor model fitted the data better both for Grit-O and for Grit-S scales (Table 2). For Grit-O, in comparison with the one-factor model (Model M1), the two-factor model (Model M2) showed better model fit. Similarly, for Grit-S, the two-factor model also fitted the data better than the one-factor model. Furthermore, the two-factor model of Grit-S (Table 2, Model M4) fitted data better than the two-factor model of Grit-O (Table 2, Model M2). This confirms that a model with two-factor structure fitted the data better than the unidimensional one-factor structure, and that the two-factor Grit-S model showed better model goodness of fit than the two-factor Grit-O model.

**TABLE 2:** Model fit indices.

**Model**	**Description**	***n***	**Para**	***χ*^2^**	***df***	***p***	**RMSEA**	**CFI**	**TLI**	**Model of comparison**	***χ*^2^ difference**	***df***	***p***
M1	Grit-O 1 factor	2,027	60	4,076.915	54	<0.001	0.192	0.728	0.668				
M2	Grit-O 2 factor	2,027	61	1,915.858	53	<0.001	0.132	0.874	0.843				
M3	Grit-S 1 factor	2,027	40	2,129.356	20	<0.001	0.228	0.797	0.716				
M4	Grit-S 2 factor	2,027	41	1,010.425	19	<0.001	0.160	0.905	0.859				
M5	m 4 revised	2,027	42	171.851	18	<0.001	0.065	0.985	0.977				
M5.1	Configural invariance	2,027	84	209.839	36	<0.001	0.069	0.983	0.973				
M5.2	Weak invariance	2,027	73	212.082	47	<0.001	0.059	0.983	0.980	M5.1	30.045	11	0.0016
M5.3	Strong invariance	2,027	55	276.72	65	<0.001	0.057	0.979	0.982	M5.2	69.071	18	<0.001
M5.4	Strict invariance	2,027	47	287.708	73	<0.001	0.054	0.978	0.983	M5.3	13.176	8	0.1059
M5.4a	DIF saturated	1,929	79	173.785	73	<0.001	0.038	0.989	0.987				
M5.4b	DIF invariant	1,929	67	229.606	85	<0.001	0.042	0.985	0.984	M5.4a	75.805	12	<0.001

However, the fit indices of Model M4 were still below thresholds of good model fit. An examination of the modification indices revealed that item 9 (“I finish whatever I begin”), which is intended to measure Perseverance of Effort dimension, also loads on the Consistency of Interests factor. This indicates that both Perseverance of Effort and Consistency of Interests were measured by this item. The revised two-factor Grit-S model (Model M5) with this cross-loading for both factors showed a close fit to the data (Model M5, Table 2).

The discrimination indices (factor loadings) and item thresholds (difficulty) are presented in Table 3. All item discrimination indices (loadings) were statistically significant and measured the intended grit dimension, with item 9, measuring both Perseverance of Effort and Consistency of Interests. The strengths of the discrimination indices for most items were around 0.5 or higher for most items, except for item 4 (0.351) and item 9 (0.314) for the Perseverance of Effort dimension. In terms of item thresholds for Perseverance of Effort dimension, the first three thresholds for all the four items were distributed toward the lower end distribution of the Perseverance of Effort (−2.882 to 1.264 on the standardized latent variable scale). Item 4 is the only item with a threshold measuring the latent variable just beyond 1 standard deviation above the mean. There is a lack of items measuring Perseverance of Effort on higher range of the latent variable distribution. The item overage of thresholds for items measuring Consistency of Interest was broader, ranging from −2.414 to 1.787.

**TABLE 3:** Factor loadings and threshold for revised Grit-S (m5).

**Item**	**Item description**	**Discrimination index**		**Threshold**			
		**PE**	**CI**	**1**	**2**	**3**	**4**
Grit_4	Setbacks don’t discourage me.	0.351		–2.203	–0.94	–0.181	1.264
Grit_6	I am a hard worker.	0.803		–2.882	–1.838	–0.923	0.473
Grit_9	I finish whatever I begin.	0.314	0.487	–2.414	–1.45	–0.581	0.853
Grit_12	I am diligent.	0.826		–2.657	–1.656	–0.72	0.864
Grit_2	New ideas and projects sometimes distract me from previous ones.		0.509	–1.383	0.007	0.702	1.787
Grit_5	I have been obsessed with a certain idea or project for a short time but later lost interest.		0.793	–1.966	–0.944	–0.21	1.091
Grit_7	I often set a goal but later choose to pursue a different one.		0.744	–2.091	–0.959	–0.131	1.321
Grit_8	I have difficulty maintaining my focus on projects that take more than a few months to complete.		0.844	–1.992	–0.891	–0.274	1.037
Grit_9	I finish whatever I begin.		0.487	–2.414	–1.45	–0.581	0.853

*All discrimination indices (standardized factor loadings) and thresholds are statistically significant at p < 0.001. Thresholds indicated on a standardized latent variable scale (variances of latent variables were fixed to 1). PE, perseverance of effort; *CI*, consistency of effort.*

### Measurement Invariance of Grit

Measurement invariance in relation to gender was performed over four sequential models (M5.1–M5.4), based on the factor structure of model M5. Comparisons of more-restrictive model against less-restrictive model showed minimal change in recommended fit indices in terms of RMSEA, CLI, and TLI (Table 2), supporting weak invariance (M5.2 vs. M5.1), strong invariance (M5.3 vs. M5.2), and strict invariance (M5.4 vs. M5.3). The chi-square difference tests were statistically significant, but the results should be interpreted with caution due to the large sample size in the present study.

Measurement invariance in terms of DIF (threshold) was examined for age and prior education levels in model M5.3 (baseline saturated model) and model 5.4 (comparison constrained model). Fit indices suggest minimal change in goodness of fit to data between models M5.3 and M5.4, indicating absence of DIF across age and prior education levels.

### Predictive Validity of Grit in Academic Performance

Latent correlations of the Grit-S factors and covariates were presented in Table 4 (based on model M5). The Perseverance of Effort factor was positively and moderately correlated with the Consistency of Interests factor at 0.383. In particular, Consistency of Interests rather than Perseverance of Effort was positively correlated with course credits (*r* = 0.079) and exam attempt (*r* = 0.154). Furthermore, older students, and students with higher prior education rated higher on both Perseverance of Effort and Consistency of Interests. Male students reported lower values on Perseverance of Effort and Consistency of Interests. In subsequent prediction analyses for the effect of grit on academic achievement, we controlled for the effects of age, gender, and level of previous education.

**TABLE 4:** Correlation between Grit-S factors and covariates.

**Variables**		**1**	**2**	**3**	**4**	**5**	**6**	**7**	**8**
PE	1	1							
CI	2	***0.383***	1						
Age	3	***0.087***	***0.164***	1					
Male^*a*^	4	***−0.099***	***−0.060***	**0.025**	1				
Prior education	5	***0.1***	***0.145***	***0.145***	***−0.034***	1			
Course credit	6	0.025	**0.079**	***−***0.051	**0.038**	***−***0.029	1		
Course grade	7	0.051	***−***0.028	0.041	***0.047***	***0.092***	***0.125***	1	
Attempted an exam	8	***−***0.03	***0.154***	***−0.166***	***−***0.013	**0.069**	0.000	0.000	1

*^*a*^Binary variable, correlation is based on biserial correlation. PE, perseverance of effort; CI, consistency of effort. Values with *p* ≤ 0.05 are indicated with bold font; values with *p* ≤ 0.01 are indicated with bold and italic font.*

Based on SEM analysis incorporating the two-factor Grit-S IRT measurement model (M5), the predictive validity of the revised Grit-S scale was assessed. Controlling for the effect of age, gender, and prior education, Consistency of Interests predicted significantly higher course credits (*beta* = 0.101, *p* = 0.033) and higher likelihood of attempting an exam (odds ratio = 1.436, *p* < 0.001; Table 5). While the Perseverance of Effort factor positively predicted course grade (*beta* = 0.087, *p* = 0.044), it also moderately predicted a lower likelihood to attempt an exam (*odds ratio* = 0.858, *p* = 0.015). Further analysis included interaction terms with gender and age, but the effect of grit factor was not found to be moderated by gender nor age (Table 5).

**TABLE 5:** Academic performance predicted by Grit-S factors.

**Course credit (*n* = 912)**				
**Predictors**	**Beta**	***p***	**Beta**	***p***
PE	0.015	0.725	0.197	0.178
CI	**0.101**	**0.033**	***−***0.13	0.381
Age	***−*0.070**	**0.044**	***−*0.072**	**0.016**
Male	**0.194**	**0.003**	**0.18**	**0.012**
Prior.edu	***−***0.029	0.427	***−***0.033	0.37
PE*age			***−***0.009	0.86
CI*age			0.005	0.916
PE*male			***−***0.131	0.29
CI*male			0.168	0.173

**Course grade (*n* = 912)**				
**Predictors**	**Beta**	***p***	**Beta**	***p***

PE	**0.087**	**0.044**	0.064	0.608
CI	***−***0.066	0.119	***−***0.047	0.716
Age	0.025	0.483	0.023	0.467
Male	**0.228**	**0.001**	**0.233**	**0.001**
Prior.edu	0.097	0.006	**0.092**	**0.016**
PE*age			***−***0.015	0.677
CI*age			0.007	0.843
PE*male			0.016	0.855
CI*male			***−***0.008	0.928

**Attempted an exam (*n* = 1,929)**				
**Predictors**	**OR**	***p***	**OR**	***p***

PE	**0.858**	**0.015**	0.913	0.634
CI	**1.436**	**<0.001**	**1.579**	**0.013**
Age	**0.730**	**<0.001**	**0.733**	**<0.001**
Male	0.965	0.717	0.958	0.670
Prior.edu	**1.136**	**0.008**	**1.135**	**0.008**
PE*age			0.980	0.755
CI*age			0.978	0.718
PE*male			0.956	0.724
CI*male			0.932	0.566

*All variables in the regression analysis were standardized except for gender. PE, perseverance of effort; CI, consistency of effort; OR, odds ratio representing the likelihood of attempting an exam compared to not; all variables in the regression analysis were standardized except for gender. Values with *p* ≤ 0.05 are indicated with bold font; values with *p* ≤ 0.01 are indicated with bold and italic font.*

## Discussion

### Factor Structure of Grit

The present investigation assessed the psychometric properties of the Grit Questionnaire in a sample of adult distance education students. In terms of measurement validity, the two-factor structure fits the data better than the one-factor structure, and this is the case both for Grit-O and Grit-S scales. In addition, the two-factor Grit-S model fitted the data better than the two-factor Grit-O model. The finding that the two-factor model, in particular the short version of the Grit Questionnaire, fits the data better than the unidimensional model confirmed findings from previous studies and recent debates (also see Muenks et al., 2017; Credé, 2018; Guo et al., 2019). The Consistency of Interests and Perseverance of Effort factors are separate factors with a moderate correlation, consistent with what has been reported in previous meta-analyses (Credé et al., 2017; Guo et al., 2019). This suggests that in an adult distance education student population, the internal structure of the grit construct remains similar to the measure in traditional higher education students.

Although the item “I finish whatever I begin” was intended to measure Perseverance of Effort, in the current sample, it was also found to load on the Consistency of Interests factor. This finding indicates that this item measures both factors. This may reflect on the specific characteristics of the distance education students. For example, for them being able to maintain effort may be something that is closely linked with holding the same goals of interests over a long period in order to pursue a learning program. However, further replication is necessary to confirm this is not a chance finding based on sample fluctuation.

The measurement precision of Perseverance of Effort dimension is in need of further improvement. Two items from this dimension had rather low item discrimination indices (factor loading), and furthermore, the thresholds (difficulty) of items measuring Perseverance of Effort had a rather sparse covering in the higher range of the latent variable distribution, indicating measurement precision is likely poor for individuals with high levels of Perseverance of Effort. The suboptimal psychometric property of the Perseverance of Effort dimension is reflected by the low reliability index measured by Chronbach’s alpha. In future research, more items with higher discrimination and higher threshold levels should be developed in order to improve the measurement quality of grit Perseverance of Effort dimension.

### Measurement Invariance of Grit

The psychometric properties of the Grit-S scale were found to be invariant across gender, age, and prior education. This finding is in line with previous research based on adult population, which also found measurement invariance across genders (e.g., Duckworth and Quinn, 2009; Wyszyńska et al., 2017) and educational levels comparing samples attending secondary (e.g., Areepattamannil and Khine, 2017) and/or university education (e.g., Datu et al., 2016). This indicates that the measurement properties Grit-S scale is comparable across gender, age and prior education.

### Predictive Validity of Grit in Academic Performance

The Consistency of Interests factor was positively predictive of the number of course credits as well as the likelihood to make a course attempt. This finding is in line with the previous Polish study where in adults Consistency of Interests but not Perseverance of Effort was associated with educational attainment (Palczyńska and Świst, 2018). This may be due to the specific profile of adult distance education students: first, more than 70% of the students already possessed a higher education-level degree; furthermore, many distance education students are often older than traditional higher education students and have family and/or work responsibilities. As such, the intention to pursue a distance education study might lie in interests of career advancement and/or motivation for self-improvement. For example, a student who enrolls in distance education could be motivated for career advancement via obtaining a degree (e.g., Business Administration), or out of a more intrinsic motivation such as pursuing a personal hobby in certain subject (e.g., a course on art in Italy). In this sense, promoting and maintaining personal interests and motivation might be especially important for increasing the academic performance of distance education students.

Results also showed that Perseverance of Effort factor positively predicted the course grade, but there was a negative effect on taking a course attempt. The finding regarding the positive effect on course grades is consistent with previous research based on adults and college students (e.g., Akos and Kretchmar, 2017; Muenks et al., 2017; Credé, 2018). However, the adverse effect on the likelihood to attempt a course exam is a unique finding. On the one hand, this could be due to students who invest more effort would deliberately delay the exam attempt because they feel they need to spend more time to prepare for the exam. On the other hand, this result might be biased and should be interpreted with caution, because the reliability and measurement precision are relatively low for the Perseverance of Effort dimension. Measurement errors can bias the effect coefficient thus this effect could be the result of a statistical bias (Loken and Gelman, 2017).

In sum, the results showed partial support to the predictive validity of grit, since the Consistency of Interest but not Perseverance of Effort positively predicted course credits and exam attempt, both are important steps in successfully obtaining the final degree. This in part contradicts the theoretical prediction of grit that both perseverance and passion are required to obtain success (Duckworth et al., 2007). This may be partly due to some of the issues related to grit which have in recent years been under rather intense debate regarding the validity and measurement practice (Credé, 2018, 2019; Guo et al., 2019). In the following sections, we discuss more extensively the issues related to grit construct in terms of its limitations and potential future directions.

## Strengths and Limitations

The present investigation used IRT-based psychometric and SEM analysis to evaluate the factor structure, measurement properties across gender, age, educational levels, as well as predictive validity of the grit construct measured by the Grit Questionnaire. The sample is also of reasonable size. Since the university involved is the only distance education institute for university-level higher education in Netherlands, to a degree, the current study includes the whole target population within 1 calendar year. Participants in the ALOUD study are comparable with the general population of students who normally study at this university (Moerkerke, 2014). Nevertheless, the findings are still limited due to the largely cross-sectional nature of the data. There is likely a reciprocal relationship between Perseverance of Effort and/or Consistency of Interests and academic performance. Higher scores on either or both factors may lead to better academic performance, and better performance could further reinforce the value of being gritty. Also, we did not test the reliability of the Grit scale over multiple assessment occasions; therefore, it is still unknown whether the measurement properties hold constant across time. Longitudinal data is required in order to establish possible causal effects between academic performance and grit, as well as to assess test-retest reliability of the Grit Questionnaire. Furthermore, the present study did not compare the psychometric equivalency of participants from socioeconomic background, nor the original English version of the Grit Questionnaire with the translated Dutch version of the Grit Questionnaire. These limitations from the current investigation would be important aspects to be addressed by future research.

Although the construction of the Grit Questionnaire is relatively recent, its connotation bears a similarity to already existing constructs (Muenks et al., 2017; Credé, 2018; Steinmayr et al., 2018). In particular, grit measured by the Grit Questionnaire has been noted to share considerable overlap with the personality trait conscientiousness, a disposition of being able to plan, organize, and persist to achieve long-term goals (Duckworth et al., 2007; Duckworth and Quinn, 2009), as well as self-control, the capacity to regulate attention, emotion, and behavior in the presence of temptation (Duckworth and Gross, 2014). However, Duckworth has suggested that grit differentiates from the personality trait conscientiousness by the focus on “effort and interest over time” and showed that grit independently predicts academic performance above and beyond personality and similar traits such as self-control (Duckworth et al., 2007; Duckworth and Quinn, 2009).

Other predictive factors of achievement that have been looked at in comparison with grit include effort regulation, self-efficacy (Muenks et al., 2017; Usher et al., 2018), self-regulated learning (Wolters and Hussain, 2015; Xu et al., 2020), and self-esteem (Weisskirch, 2018), all of which are also constructs theorized to influence academic performance (Schunk and Greene, 2018). However, these constructs are often measured in a domain specific way (Bong, 2001). For example, questionnaire items assessing self-efficacy often specifically refer to the academic subject (e.g., maths or reading). Self-efficacy in maths can be more highly correlated with academic performance in maths compared with academic performance in reading. The Grit Questionnaire, on the other hand, measures a rather general concept thus lacking a contextual focus which may attenuate the predictive validity for domain specific performance. It has been suggested that the limited predictive effect of grit might be due to the fact that the Grit scale is a domain general construct, therefore lacking specificity directly relevant for academic performance. Recent research in measurement of grit has proposed and developed instruments of grit that measure different domains, academic and nonacademic (Schmidt et al., 2017; Cormier et al., 2019). It has been shown that grit, when measured specific to a domain, is more highly correlated with performance in the corresponding domain (e.g., academic grit is more highly correlated with academic performance). In future adult distance education studies of grit, academic grit can be assessed to pin-point the predictive effect of grit on academic performance.

## Conclusion and Implications

To conclude, the current study showed that the Consistency of Interests rather than Perseverance of Effort is a predictor of academic performance in adult distance education students, and that the measurement precision of the Perseverance of Effort dimension needs to be improved. Further research in this population needs to refine the measurement of the Grit Questionnaire in order to better understand the role of grit in the academic success of adult distance education students, and what further factors might explain the relationship between Consistency of Interest and academic performance.

There are intervention studies that have shown that certain personality traits similar to Perseverance of Effort, such as conscientiousness, can be responsive to interventions (Roberts et al., 2017). Nevertheless, in most of previous research, the effect size of grit on academic achievement was rather modest, indicating potentially limited efficacy if an intervention is put into practice. Future studies in adult distance education students should explore the extent to which the grit construct can uniquely predict academic performance in order to fully establish the usefulness of this instrument to the previously established constructs that also predict academic performance. Until then, caution should be taken regarding designing interventions to “raise” grit in adult distance education students as a means to promote academic performance.

## Data Availability Statement

The data analyzed in this study is subject to the following licenses/restrictions: the data used for the current article is part of a larger datasets that securely stored on an institutional server. It is thus not possible to for the data to be publicly available from open servers free to download. Requests to access these datasets should be directed to RG, renate.degroot@ou.nl.

## Ethics Statement

The studies involving human participants were reviewed and approved by the Ethical Committe of the Open University of the Netherlands. The patients/participants provided their written informed consent to participate in this study.

## Author Contributions

KX designed and performed the analysis, and wrote the manuscript. CM, HG, JN, and RG provided critical input on the design and substantially improved the manuscript. All authors contributed to the article and approved the submitted version.

## Conflict of Interest

The authors declare that the research was conducted in the absence of any commercial or financial relationships that could be construed as a potential conflict of interest.
